# Cannabinoid CB1 receptors regulate salivation

**DOI:** 10.1038/s41598-022-17987-2

**Published:** 2022-08-19

**Authors:** Kelsey Andreis, Jenna Billingsley, Kian Naimi Shirazi, Jim Wager-Miller, Clare Johnson, Heather Bradshaw, Alex Straiker

**Affiliations:** 1 Department of Psychological and Brain Sciences, Gill Center for Biomolecular Science, Program in Neuroscience, Indiana University, Bloomington, IN 47405 USA; 2 Indiana University, 1101 E 10th St, Bloomington, IN 47401 USA

**Keywords:** Neuroscience, Cellular neuroscience, Peripheral nervous system

## Abstract

Saliva serves multiple important functions within the body that we typically take for granted, such as helping prepare food for swallowing and defense against oral pathogens. Dry mouth is a primary symptom of Sjӧgren’s syndrome and is a side effect of many drug treatments. Cannabis users frequently report dry mouth, but the basis for this is still unknown. If the effects occur via the endogenous cannabinoid signaling system, then this may represent a novel mechanism for the regulation of salivation. We examined expression of cannabinoid CB1 receptors in submandibular salivary gland using immunohistochemistry and tested regulation of salivation by THC and cannabinoid-related ligands. We now report that CB1 receptors are expressed in the axons of cholinergic neurons innervating the submandibular gland. No staining is seen in submandibular gland epithelial cells (acinar and ductal), or myoepithelial cells (MECs). Treatment with THC (4 mg/kg, IP) or the cannabinoid receptor agonist CP55940 (0.5 mg/kg) reduced salivation in both male and female mice 1 h after treatment. CBD had no effect on its own but reversed the effect of THC in a concentration-dependent manner. Neither the CB1 receptor antagonist SR141716 (4 mg/kg) nor the CB2-selective agonist JWH133 (4 mg/kg) had an effect on salivation. We also found that fatty acid amide hydrolase (FAAH), the enzyme that metabolizes the endocannabinoid anandamide and related lipids, regulates salivation. Salivation was reduced in FAAH knockout mice as well as mice treated with the FAAH blocker URB597 (4 mg/kg). URB597 had no effect in CB1 knockout mice. FAAH protein is detected intracellularly in acinar but not ductal epithelial cells. In lipidomics experiments, we found that FAAH knockout mice chiefly had elevated levels of acylethanolamines, including anandamide, and reduced levels of acyglycines. Our results are consistent with a model wherein endocannabinoids activate CB1 receptors on cholinergic axons innervating the submandibular gland. THC likely acts by plugging into this system, activating CB1 receptors to reduce salivation, thus offering a mechanism underlying the dry mouth reported by cannabis users.

## Introduction

The salivary glands are a group of exocrine glands that produce the saliva in the mouth, largely under neuronal regulation from the brainstem^1^. When the salivary glands malfunction the resultant insufficiency of salivation can cause several forms of xerostomia or dry mouth. Estimates of the prevalence of chronic xerostomia vary but certainly affects millions of Americans^2^. The difficulty swallowing associated with chronic xerostomia can substantially lower quality of life and health and insufficiency of saliva is accompanied by risk of serious complications including oral infections^3^. Women and the elderly are more likely to suffer from chronic xerostomia, the latter in part from side effects of polypharmacy^4,5^.

CB1 receptors are part of a family of G protein-coupled lipid receptors (GLRs) that are activated by related endogenous lipids, chiefly acylglycerols, acylethanolamines, and acylglycines. These receptors often play complementary roles in regulation of peripheral physiological systems. For instance, we have found that GPR119, GPR18, and CB1 differentially regulate ocular pressure^6–9^ while the chemotaxic migration of corneal epithelial cells is differentially controlled by GPR18, CB1 and CB2^10–12^. Most of these receptors are activated by THC, the chief euphoric component of cannabis, but CB1 receptors are considered the most likely to mediate CNS effects of cannabis. Cannabis users frequently report dry eye and dry mouth (e.g., Ref.^13^). We have recently shown that CB1 receptors regulate tearing, likely via inhibition of neuronal parasympathetic inputs to the lacrimal gland^14^. Moreover, this regulation is sex-dependent—at least in mouse—with opposing effects on tearing in males and females. As exocrine glands that are under opposing parasympathetic/sympathetic control, salivary and lacrimal glands have much in common, including regulation by the same brainstem nucleus (superior salivatory nucleus)^1,15^. Though potential salivary gland roles for some of these receptors have been reported, such as Korchinsky et al. that implicates GPR55 in salivary gland development^16^, the remaining receptors have seen limited (CB1/CB2)^17–24^ or no study in this context. The current project was conceived to test whether and how CB1 receptors might regulate salivary gland function.

## Methods

### Animals

Experiments were conducted at the Indiana University campus. All mice used for experiments were handled according to the guidelines of, and approved by, the Indiana University IACUC animal care committee. Experiments are reported in accordance with ARRIVE guidelines. Adult mice (both sexes, age 3–8 months) were kept on a 12 h (06:00–18:00) light dark cycle and fed ad libitum. C57BL/6J (C57), CD1 and CB1 KO mice were kindly provided by the laboratory of Dr. Ken Mackie (Indiana University, Bloomington IN) or purchased from Jackson Laboratories. Conventional CB1 null mice (CB1^−/−^) were originally received from Dr. Catherine Ledent (Catholic University, Leuven)^25^. Conventional FAAH null mice (FAAH^−/−^) were originally received from Dr. Benjamin Cravatt (Scripps Research Institute, La Jolla, CA, USA)^26^.

### Method of measuring salivation

**Figure 1:**
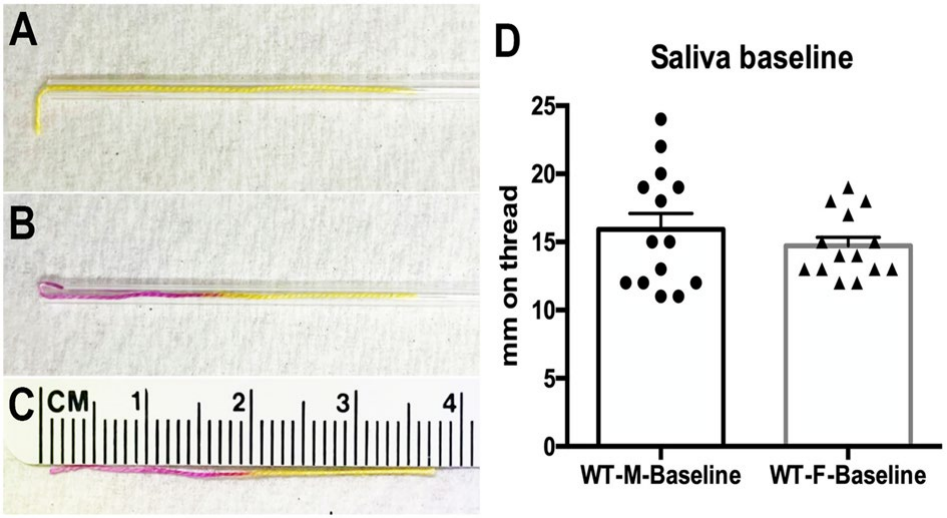
Capillary based method for measuring basal salivation in mice. (**A**) Phenol red-coated thread is inserted into a fire-polished glass capillary with 3 mm extending outside the opening. This is then placed under the tongue of an isoflurane anesthetized mouse for 10 s. (**B**) Saliva travels up the thread, discoloring it. (**C**) The distance traveled in mm is taken as a measure of basal saliva. (**D**) Sample baseline data for WT males and females shows that basal levels are similar (n = 14 per condition).

To measure basal salivation in mice we developed a minimally invasive method that makes use of phenol red coated threads (see Fig. 1). Developed initially for ophthalmic use, these threads are discolored by the pH of tears and saliva, which rapidly travel along the thread allowing the distance traveled along the thread to be taken as a measure of tearing/salivation. To allow for consistent placement in the oral cavity, a thread is inserted into a glass capillary (fire-polished to prevent oral injury), leaving 3 mm of thread outside the opening of the capillary. The tip of this glass capillary is then placed under the tongue of an isoflurane anesthetized mouse for 10 s. The induction of anesthesia is rapid (< 3 min) and mice quickly recover from the anesthesia with first signs of recovery ~ 1 min after removal from isoflurane. Animals are ambulatory within ~ 5 min and so spend the bulk of the time between measurements active in their home cages. Drug effects at 1 h are compared to control animals at 1 h using an unpaired t-test unless otherwise stated. For experiments that test the effect of drug treatments, animals are injected with drugs intraperitoneally (IP) 1 h before measurement of salivation. Drug concentrations were determined based on EC50/IC50 values and/or previously reported concentrations in vivo (E.g. Ref.^27^ for SR141716).

### Immunohistochemistry

For immunohistochemistry, submandibular glands were fixed in 4% paraformaldehyde for 45 min at 4 °C, then placed sequentially in 10% and 30% sucrose in PBS overnight before being suspended in OCT (Thermo Fisher Scientific, Waltham, MA, USA) in a 15 mL plastic test tube, then submerged in cold (− 80 °C) methanol. Our immunohistochemical methods have been published previously (e.g. Ref.^28^), but briefly: fixed SMG tissue was sectioned on a Leica cryostat (Leica Microsystems, Wetzlar, Germany), then sections were mounted on Superfrost Plus slides (Thermo Fisher). Slides were blocked with BSA, followed by treatment with primary antibodies (in PBS, saponin, 0.2%) for 1–2 days at 4 °C. See Table 1 for list of primary antibodies. In cases where secondary antibodies were required, a second staining with secondary antibody (~ 4 h at RT) was done after washing off the primary antibody. Appropriate secondary antibodies were labeled with the Alexa488, Alexa594, or Alexa647 (Thermo Fisher). Slides were then mounted with mounting media containing 4′,6-diamidine-2′-phenylindole dihydrochloride (DAPI) to visualize nuclei (Fluoromount, Sigma-Aldrich, St. Louis, MO, USA). Images were acquired with a Leica TCS SP5 (Leica Microsystems)**.** Images were processed using FIJI (vsn 2.3.0/1.53q, available at https://imagej.net/Fiji/downloads) and/or IMARIS Image Analysis Software (BITPLANE, Oxford Instruments) and/or Photoshop vsn. 21.2 (Adobe Inc., San Jose, CA, USA) software. Images were modified only in terms of brightness and contrast.

**Table 1:** Antibodies/primary labels used in this study.

Target	Host	Source	Cat#	Lot#	Concentration
FAAH	Rabbit	Synaptic systems	SA0666	NA	1:300
CB1	Guinea pig	Frontier	CB1-GP-Af530	NA	1:300
MAGL	Rabbit	Frontier	MGL-Rb-Af200	AB_2571798	1:300
NAPE-PLD	Guinea Pig	Frontier	NAPE-PLD-GP-Af720	AB112350	1:300
Choline acetyl transferase (ChAT)	Goat	Millipore	AB144P	2,211,015	1:150
Phalloidin-488, 594	N/A	ThermoFisher	A12379	1,749,905	1:300
FAAH (Western)	Rabbit	Ken Mackie	NA	NA	1:1000

### Western Blot methods

Salivary glands were harvested and quick frozen on dry ice. Tissue punches (4 mm) were made from the frozen tissue and quick homogenized in RIPA buffer [50 mM Tris, pH8.0, 150 mM NaCl, 1% Tx-100, 0.1% SDS, 0.05% sodium deoxycholate and 1 × HALT (Thermo Fisher Scientific, #78440)] and centrifuged. Supernatants were run on a 4–12% NuPage gel (Thermo Fisher Scientific, #NP0323BOX) and transferred to nitrocellulose. Blots were stained and quantitated with a reversible dye (Li-Cor, # 926-11011), blocked for 1 h in blocking buffer (Li-Cor, #927-70001), and then incubated at 4 °C overnight with rabbit anti-FAAH antibody (Mackie Lab; 1:1000) diluted in blocking buffer. They were then washed four times 15 min with TBS-T and incubated at room temperature in donkey anti-rabbit IgG secondary antibody (Li-Cor, #926-32212) for 1 h and washed again. An Odyssey near-IR imager was used to collect blot images. ImageJ software was used to analyze specific band intensities and total protein load. Specific bands were normalized to total protein loaded.

### Lipidomics methods

SMG of FAAH KO mice were dissected and rapidly flash frozen in liquid N2 and stored at − 80 °C. Lipid extractions and HPLC/MS/MS analysis was performed as previously described^29,30^. Briefly, 5 µL of 1 µM deuterium labeled AEA (d8AEA), 2 mL of 100% HPLC-grade methanol and samples were incubated on ice in the dark for 2 h before being suspended by sonication and centrifuged at 19,000*g*, 20 °C, for 20 min. Supernatants were decanted into 7 mL of water to create a ~ 25% organic solution, which was partially purified on C18 solid phase extraction columns and lipids were eluted with 1.5 mL of 3 different methanolic concentrations (65, 75, and 100%), then stored at – 80 °C until analyzed by HPLC/MS/MS. Elutions were screened for 80 compounds (see Supplementary data for complete list) using an Applied Biosystems API 3000 triple quadrupole mass spectrometer with electrospray ionization (Foster City, CA, USA) in multiple reaction monitoring (MRM) mode as previously described^29,30^. Statistical analyses were completed in SPSS Statistics 27 (IBM). One-way ANOVAs followed by Fisher's Least Significant Difference post-hoc analyses were used to determine statistical differences between the average concentration of an analyte measured in WT verses KO SMG. Statistical significance for all tests was set at p < 0.05, and trending significance at 0.05 < p < 0.10. Descriptive and inferential statistics were used to create heatmaps for visualizing changes in lipids^29,30^. Briefly, the direction of changes in the treatment group compared to vehicle are depicted by color, with green representing an increase and orange representing a decrease. Level of significance is shown by color shade, wherein p < 0.05 is a dark shade and 0.05 < p < 0.1 is a light shade. Direction of the change compared to vehicle is represented by up (increase) or down (decrease) arrows. Effect size is represented by the number of arrows, where 1 arrow corresponds to 1–1.49-fold difference, 2 arrows to a 1.5–1.99-fold difference, 3 arrows to a 2–2.99-fold difference, 4 arrows a 3–9.99-fold difference, and 5 arrows a difference of tenfold or more. To calculate the effect size of a lipid that was significantly higher in the treatment group, the average concentration of the drug group is divided by the average concentration of the vehicle group. To calculate the effect size of a compound that is significantly lower in the treatment group, the same calculation is conducted, except the inverse of the resulting ratio is used to represent a fold decrease.

### Drugs

THC and CBD were obtained through the NIDA Drug Supply Program. CP55940, URB597, JWH133, and SR141716 were obtained from Cayman Chemical (Ann Arbor, MI, USA). Drug concentrations were determined based on available literature for in vivo studies (e.g. Ref.^31^ for CP55940).

## Results

### CB1 receptors are expressed in submandibular gland of the mouse.

**Figure 2:**
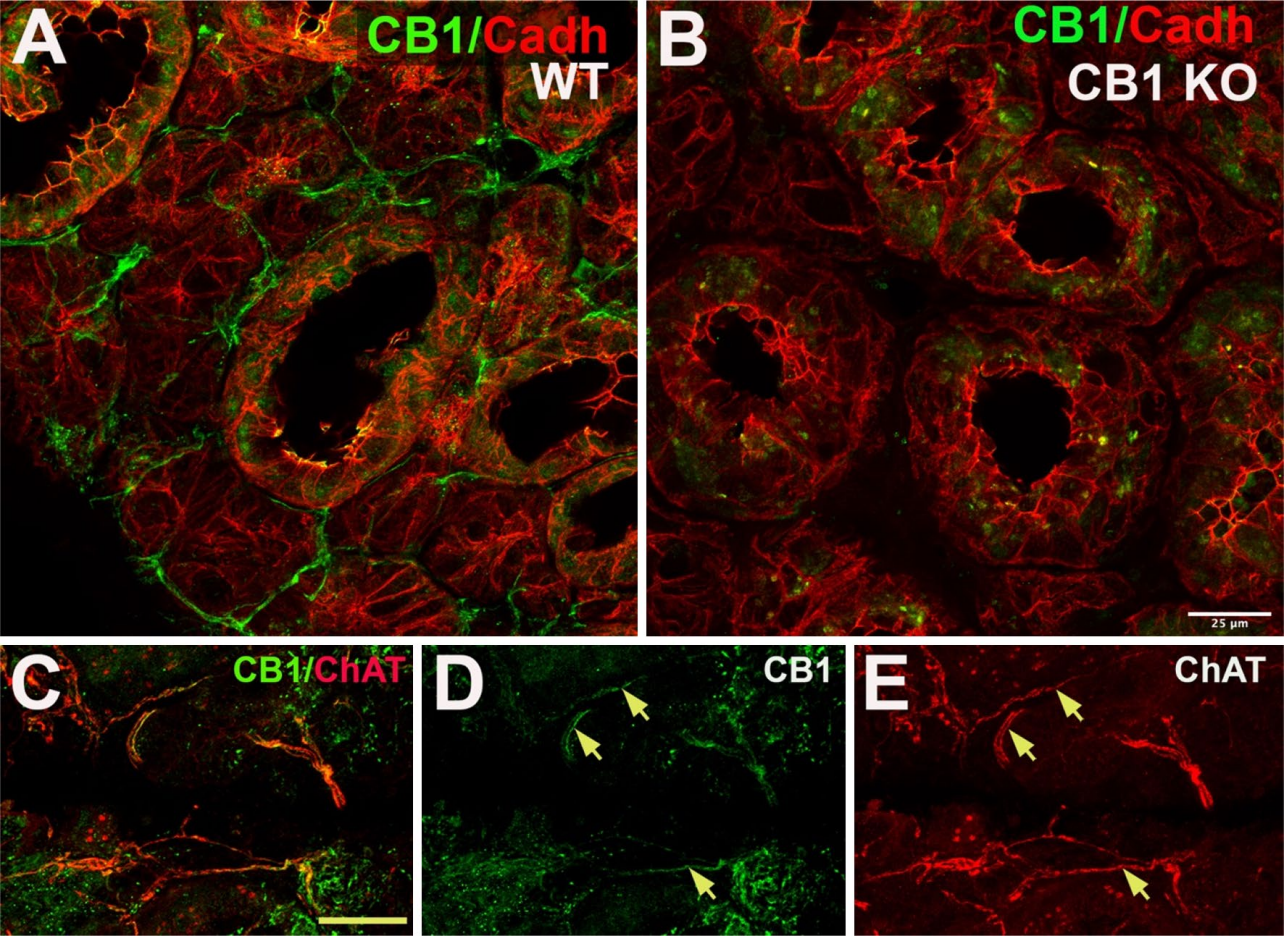
CB1 protein is expressed in a subset of cholinergic axons in submandibular gland. (**A**,**B**) Left panel shows CB1 (green) and cadherin (red) in wild type (WT) mouse submandibular gland. Right panel shows that CB1 staining in axon-like processes is absent submandibular gland from CB1 knockout mouse. (**C**) Double staining in submandibular gland of the mouse shows expression of CB1 (green) and choline acetyl transferase (ChAT, red), a marker for cholinergic axonal inputs. (**D**,**E**) CB1 (green) staining is seen in processes of ChAT-positive axons (arrows). Scale bar: (**A**,**B**) 25 µm; (**C**–**E**) 40 µm. Images processed using Adobe Photoshop vsn. 21.2 and FIJI (vsn 2.3.0/1.53q, available at https://imagej.net/Fiji/downloads).

Using immunohistochemistry, we tested CB1 receptor expression in the submandibular gland (SMG) of the mouse (Fig. 2A). Specificity of the staining was confirmed in SMG from CB1 knockout mice (CB1^−/−^) (Fig. 2B). We found that myoepithelial and acinar cells had no or little CB1 protein expression, while expression could be seen in axon-like processes (Fig. 2A,C). Costaining with antibodies against choline acetyl transferase (ChAT), a marker for cholinergic neurons showed frequent co-staining with CB1 (Fig. 2C–E), suggesting that CB1 is present on parasympathetic afferents.

### THC and CB1 agonist CP55940 alter basal saliva volume in male and female mice via activation of cannabinoid CB1 receptors

**Figure 3:**
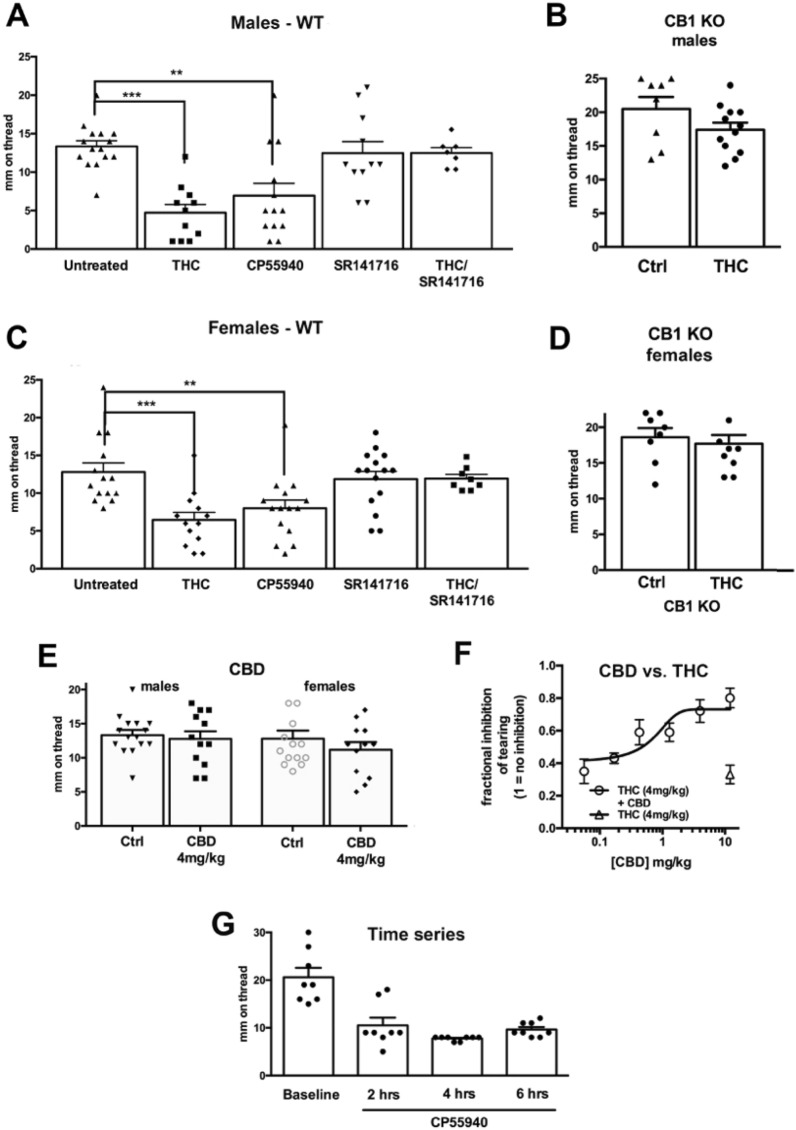
CB1 receptors regulate basal salivation. In male (**A**) and female (**C**) mice, THC (mg/kg, IP) or CP55940 (0.5 mg/kg) reduced basal salivation 1 h after treatment. The CB1 antagonist SR141716 (4 mg/kg) had no effect. (**B**,**D**) THC had no effect in CB1 knockout males (**B**) or females (**D**). (**E**) CBD (4 mg/kg) had no effect on salivation in males or females. (**F**) CBD reversed the effects of THC in a concentration-dependent manner. (**G**) Effect of CP55940 persists for at least six hours. **p < 0.01; ***p < 0.005, one-way ANOVA with Dunnett’s post-hoc test vs. control.

Salivation is subject to regulation by multiple internal and external factors. *Basal salivation* is the salivation present under normal conditions, while salivation induced by an irritating stimulus is referred to as *reflex salivation*. We tested the consequence of THC treatment on basal salivation since this most closely approximates the conditions under which cannabis users would experience altered salivation. Male mice treated with THC (4 mg/kg, IP) showed a significant reduction in saliva volume relative to WT controls. Figure 3A, Control salivation volume (mm on phenol red thread ± SEM): 13.3 ± 0.7, n = 13; THC-treated: 4.7 ± 1.1, n = 11; ***p < 0.01, 1-way ANOVA with Dunnett’s post hoc vs. WT control), consistent with the reduced salivation seen in cannabis-exposed human subjects. Similarly, the potent CB1 receptor agonist CP55940 (0.5 mg/kg) also lowered saliva volume in WT (Fig. 3A; CP55940 (mm ± SEM): 6.9 ± 1.6, n = 13; 1-way ANOVA (F(4,58) = 11.0, p < 0.0001); Dunnett’s post hoc vs. WT control, **p < 0.01 for CP55940; ***p < 0.005 for THC, NS for SR141716). This was reversed by cotreatment with the CB1 receptor antagonist SR141716 (4 mg/kg) (Fig. 3A, SR141716/THC (mm ± SEM): 12.5 ± 0.7, n = 7, NS, as above). SR141716 did not alter salivation in male wild type mice on its own (Fig. 3A, SR141716 (mm ± SEM): 12.5 ± 1.5, n = 11, NS, as above). The effect of CP55940 on salivation in males lasts at least six hours (Fig. 3G). Treatment of CB1 knockout mice with THC did not alter basal salivation (Fig. 3B, CB1 KO control (mm ± SEM): 12.4 ± 1.1, n = 9; THC in CB1KO: 13.6 ± 1.8 n = 9; NS, p < 0.60, unpaired t-test vs. CB1 KO untreated control). Because CP55940 is a non-selective agonist for CB1/CB2 receptors we also tested whether the CB2 agonist JWH133 (4 mg/kg, IP) altered salivation, finding that it did not (data not shown, males (mm ± SEM): 12.6 ± 0.8, n = 8; females: 13.5 ± 1.3, n = 11; NS, p = 0.55 for males, p = 0.71 for females, unpaired t-test vs control).

In female mice treatment with THC or CP55940 also reduced saliva levels (Fig. 3C, WT saliva volume (mm ± SEM): 12.8 ± 1.1, n = 14; THC-treated: 6.5 ± 1.0, n = 13; CP-treated: 8.0 ± 1.0, n = 15; 1-way ANOVA (F(4,67) = 10.4, p < 0.0001); Dunnett’s post hoc vs. WT control, **p < 0.01 for CP55940; ***p < 0.005 for THC, NS for SR141716). As in males, the effect of THC was blocked by the CB1 receptor antagonist SR141716 (Fig. 3C, SR141716/THC (mm ± SEM): 11.9 ± 0.5, n = 8, NS, as above) and SR141716 did not alter salivation in females on its own (Fig. 3C, SR141716 in WT females: 11.9 ± 1.0, n = 15, NS as above). Treatment of the female CB1 knockout mice with THC did not alter basal salivation (Fig. 3D, CB1 KO control (mm ± SEM): 18.6 ± 1.3, n = 8; THC in CB1 KO: 17.7 ± 1.2, n = 8; NS, unpaired t-test vs. KO control, p = 0.60).

### CBD does not alter salivation but reverses THC effects in a concentration-dependent manner

In separate experiments, treatment with the phytocannabinoid cannabidiol (CBD, 4 mg/kg IP) did not alter salivation in males or females [Fig. 3E, CBD in males (mm ± SEM): 12.8 ± 1.1, n = 12; CBD in females: 11.2 ± 1.1, n = 12; NS, unpaired t-test vs. respective WT control, p = 0.65 (males), p = 0.34 (females)]. We and others have reported that CBD reverses the effects of THC, likely as a CB1 allosteric modulator^9,32^. Consistent with this, we found that when male mice were treated with a combination of CBD and THC, the observed effect of THC was diminished in a concentration-dependent manner (Fig. 3F; IC50 (95% CI): 4.0 mg/kg (0.07–68)).

### Blockade or deletion of the acylethanolamine-metabolizing enzyme FAAH reduces salivation

**Figure 4:**
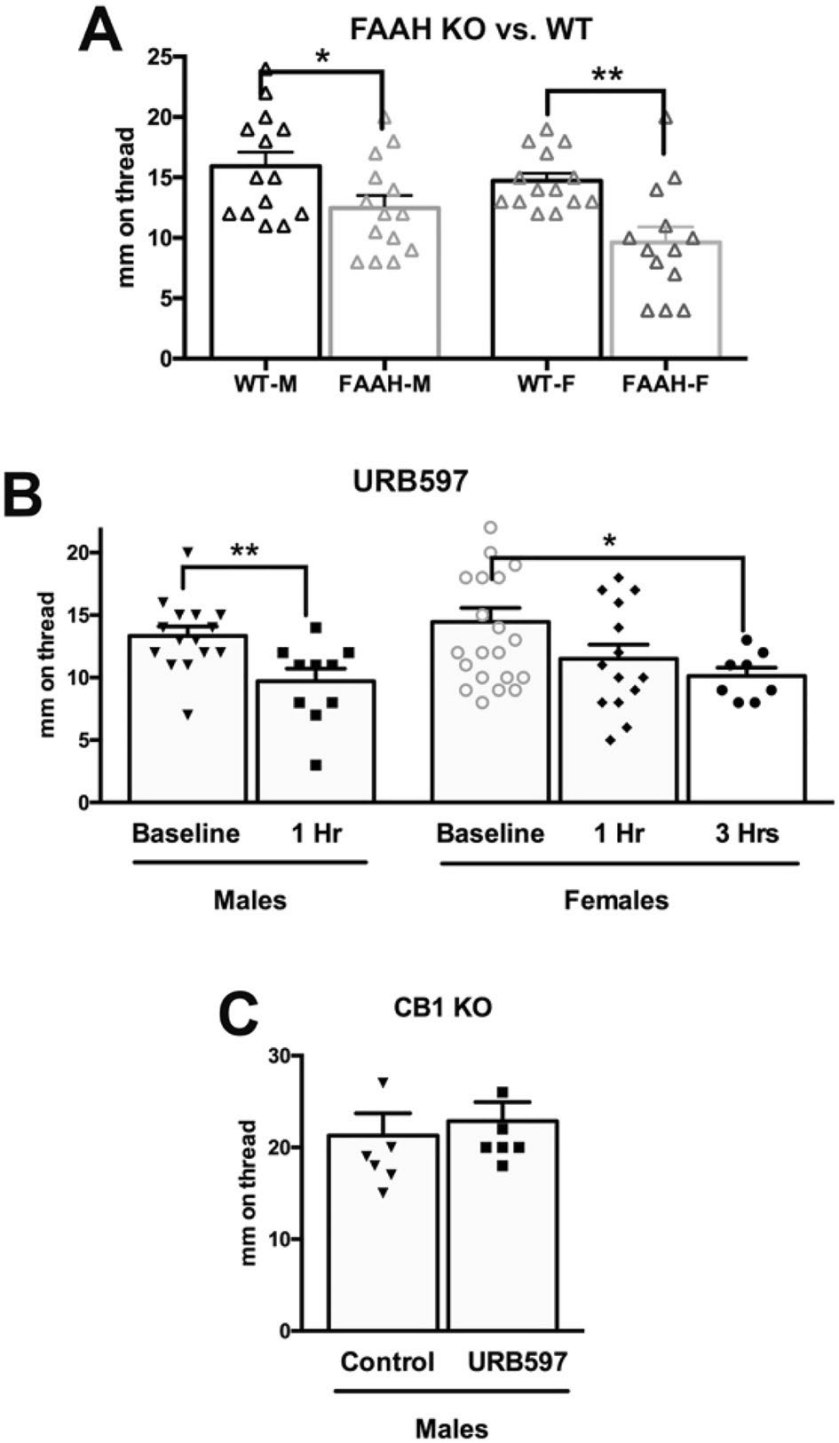
FAAH deletion and blockade reduces salivation. (**A**) Male and female knockout mice saw lower baseline salivation. (**B**) Treatment with the FAAH blocker URB597 (4 mg/kg) reduced salivation at 1 h in males and at 3 h in females. (**C**) URB597 did not alter salivation in male CB1 knockout mice. *p < 0.05, *p < 0.01 unpaired t-test.

There are two canonical endogenous CB1 receptor ligands, anandamide and 2-arachidonoyl glycerol (2-AG). However, additional endogenous lipids have been shown to also have robust activity at CB1 including the anandamide congeners, (*N*-acyl ethanolamines; NAEs), docosahexaenoyl ethanolamine and linoleoyl ethanolamine ^33,34^. Importantly, oxygenated metabolites of polyunsaturated NAEs have been shown to have activity at CB1 receptors that was more efficacious than CP55940^33^ and that blunted seizure activity in a CB1-dependent manner^34^. Each of these endogenous lipids are tonically present throughout the body, though their levels are regulated by enzymatic activity. NAEs, including anandamide, are metabolized by fatty amide hydrolase (FAAH^26^), We have shown that many additional lipids are also regulated by FAAH by illustrating dramatic changes in the lipidome in FAAH KO mice^7^ and with the FAAH inhibitor, URB597^35^. Here we tested for altered salivation in knockout mice for FAAH. We found that in both males and females the baseline saliva levels were lower in FAAH knockout mice (Fig. 4A, baseline saliva (mm on thread) in males: 12.5 ± 1.0, n = 14; in females: 9.6 ± 1.3, n = 13; *p < 0.05, **p < 0.01 by unpaired t-test; p = 0.035 for males; p = 0.001 for females). We also tested whether the FAAH inhibitor URB597 (4 mg/kg) would similarly reduce salivation after intraperitoneal injection. We found that salivation was reduced in males but not in females at one hour (Fig. 4B, saliva (mm on thread) after URB597 in males: 9.7 ± 1.0, n = 10; in females: 11.5 ± 1.1, n = 14; **p < 0.01 by unpaired t-test; p = 0.0072 for males; p = 0.08 for females). However, when salivation in females was tested 3 h after treatment, the drop was statistically significant (Fig. 4B, saliva (mm on thread) after URB597: 10.1 ± 0.67, n = 8; p = 0.03 by unpaired t-test). Our working hypothesis is that FAAH deletion/inhibition increases NAE levels and that these NAEs then act on CB1 receptors to reduce signaling that reduces salivation. If so, then URB597 should not reduce salivation in CB1 knockout mice. Consistent with this, we found that salivation in male CB1 KO mice was unaltered by treatment with URB597 (Fig. 4C; saliva (mm on thread) baseline: 21.3 ± 2.4; after URB597: 22.9 ± 2.1, n = 7; p = 0.63 by paired t-test).

### FAAH protein is expressed in acinar cells of the SMG

**Figure 5:**
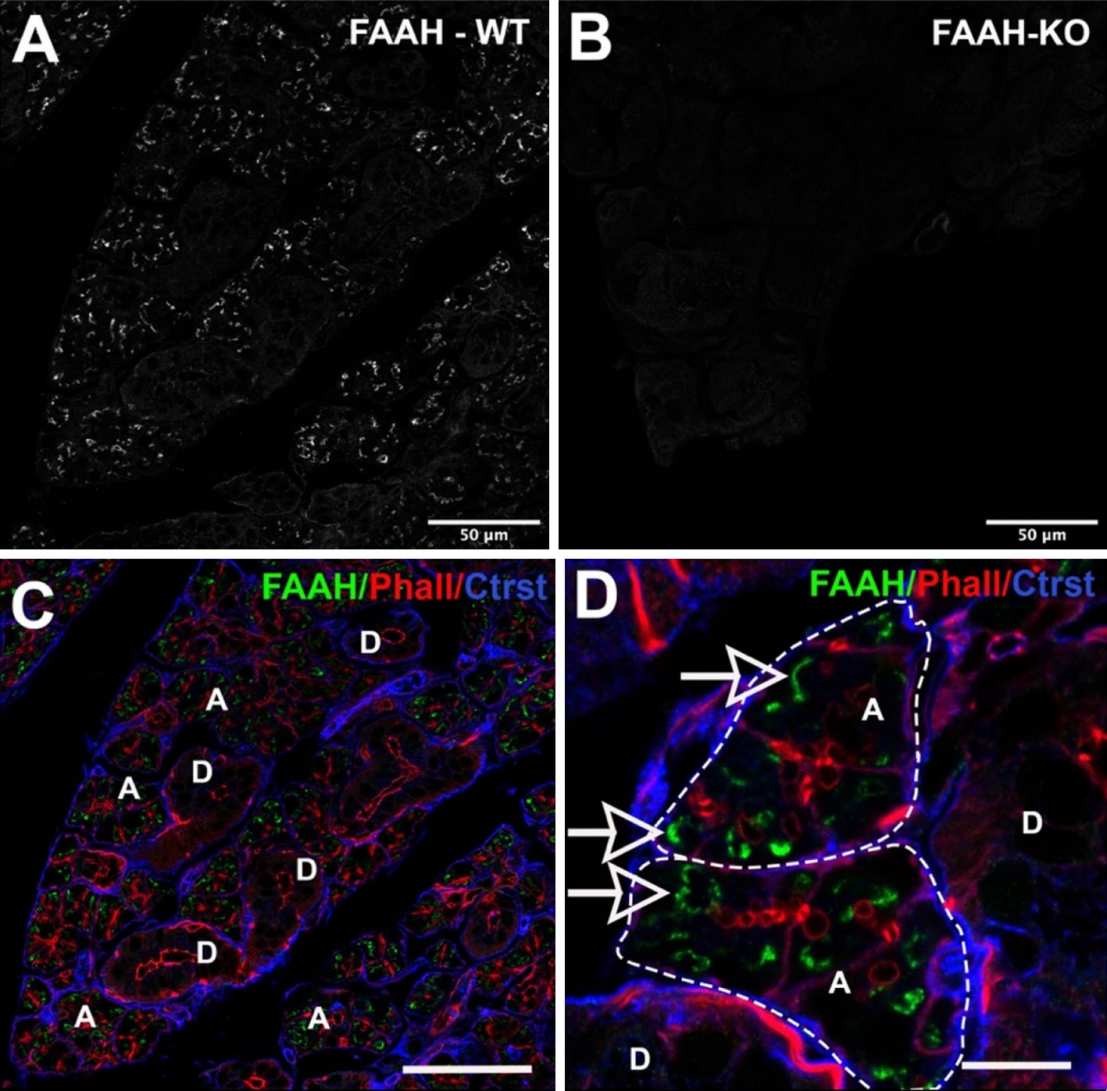
FAAH protein expression in acini of submandibular gland. (**A**,**B**) FAAH expression in (**A**) WT and (**B**) FAAH knockout submandibular gland. (**C**) Triple-stain from (**A**) shows FAAH (green) relative to phalloidin (red) and a counterstain that outlines acini. FAAH is seen in acini “A” but not ducts “D”. (**D**) Higher magnification image shows FAAH staining in acini “A”, outlined but not ducts “D”. Scale bars: (**A**–**C**) 50 µm; (**D**) 15 µm. Images processed using Adobe Photoshop vsn. 21.2 and FIJI (vsn 2.3.0/1.53q, available at https://imagej.net/Fiji/downloads).

**Figure 6:**
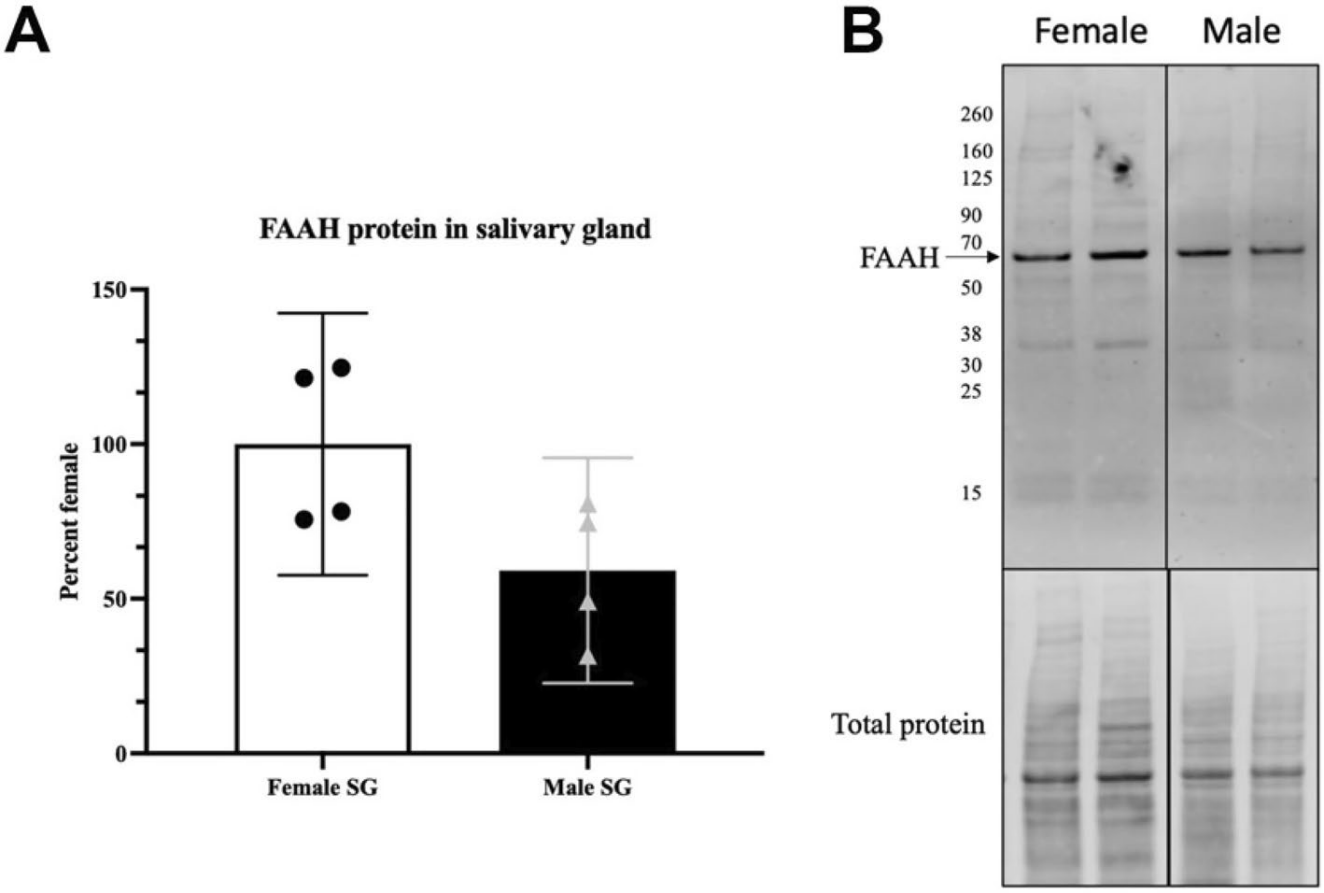
FAAH protein in male vs. female submandibular gland. (**A**) FAAH protein levels in males and females (p = 0.058, student’s t test, n = 4). (**B**) A protein band is detected at the expected molecular weight (~ 63 kDa). Full blot in Supplementary Fig. S1.

To determine where the FAAH protein is expressed we used immunohistochemistry. FAAH protein has previously been reported to localize to intracellular structures such as smooth endoplasmic reticulum and the membranes of mitochondria^36^. Consistent with this, we found that FAAH protein is expressed intracellularly within acinar cells (Fig. 5A,C–D) but not in ductal cells. Staining is absent in SMG from FAAH knockout mice (Fig. 5B). The actin marker phalloidin labels ducts within acini (e.g. Fig. 5D). FAAH did not appear to exhibit a polarized distribution within acinar cells. We also tested whether FAAH protein levels varied by sex in the submandibular gland using a Western blot assay. We detected a FAAH band at the predicted molecular weight (63kda) and found that levels were trending towards significance with levels higher in females (Fig. 6, p = 0.058 by Student’s t-test, n = 4; Full blot in Supplementary Fig. S1).

We also tested for NAPE-PLD protein expression using a knockout-validated antibody against NAPE-PLD^37^. The most prominent NAPE-PLD protein was seen in a subset of myoepithelial cells (Supplementary Fig S3).

### FAAH knockout mice have elevated acyl-ethanolamine levels and decreased acyl-glycine levels

**Figure 7:**
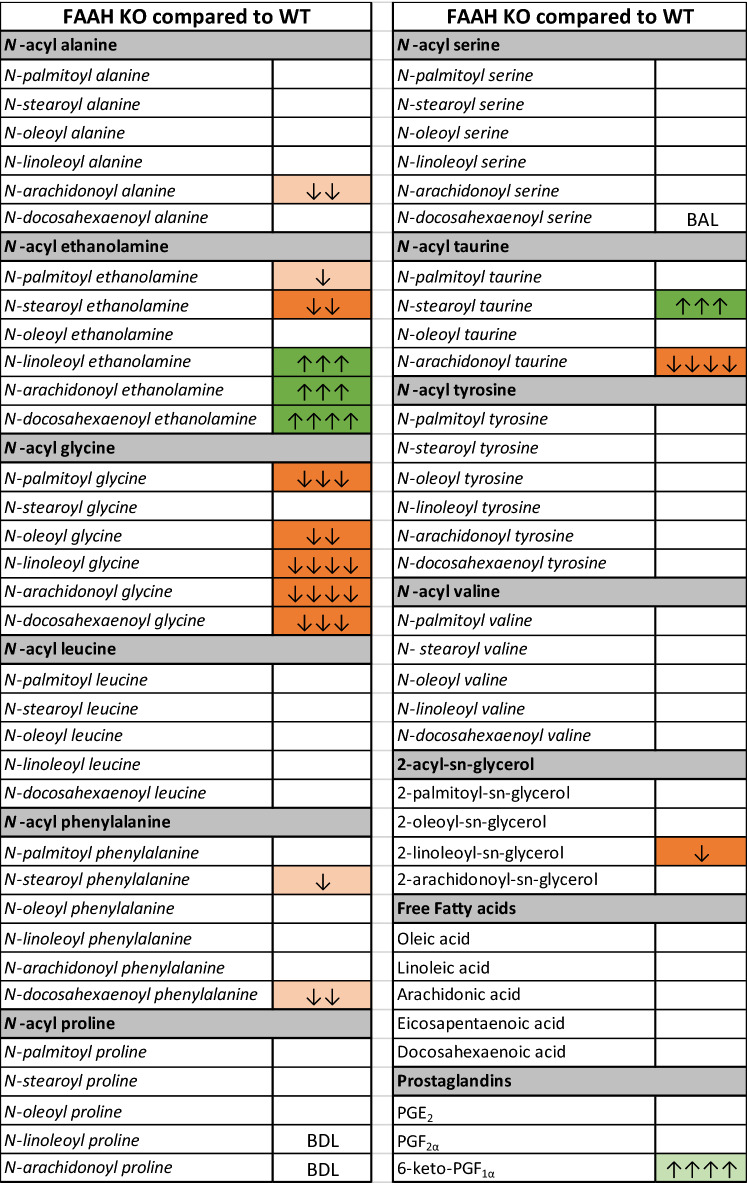
Differences in lipid levels in FAAH KO SMG compared to WT. Fold change denoted by arrows 1 = 1–1.49; 2 = 1.5–1.99; 3 = 2–2.99; 4 = 3–9.99; 5 = 10 or more. *Green* significant increases, *orange* significant decreases, *light green and orange* trending changes, *white* detected but no significant differences, *BDL* below detection limit, *BAL* below analytical limits though present in some samples.

We examined the lipidomic profile of FAAH knockout mice relative to wild type mice. Previously, we showed the FAAH KO mice had dramatically different lipid profiles in a wide range of lipids in the CNS, though some areas of the CNS were more affected than others with more changes in lipids measured in cortex than hypothalamus^38,39^ Here (Fig. 7), we tested the lipidomic profiles of SMG in male WT and FAAH KO mice. We found that levels of the NAEs, AEA, LEA, and DEA were significantly increased; OEA levels were equivalent, and levels of SEA were significantly lower in FAAH KOs. Five species of *N*-acyl glycines, the 2-AG analog, 2-linoleoyl glycerol, and arachidonoyl taurine were significantly lower.

### Blocking MAGL activity does not alter salivation

As noted above, the endocannabinoid 2-AG has also been found to mediate effects of CB1 activation, particularly in the CNS (e.g. Ref.^40^). Though FAAH appears to account for the CB1-based effects on salivation, we also tested whether blocking MAGL, the chief 2-AG metabolizing enzyme, has an impact on salivation. The MAGL blocker JZL184 (8 mg/kg, IP) did not alter salivation relative to baseline values at one hour after treatment (Supplementary Fig. S2A, salivation one hour after JZL184 (8 mg/kg) in males: 14.1 ± 0.90, n = 8; in females: 16.2 ± 1.3, n = 8, NS by paired t-test vs. pre-treatment baseline, p = 0.56 for males, p = 0.16 for females). We also tested for MAGL protein expression with an antibody that we have previously used with success in the anterior eye^6^ but did not detect MAGL protein expression in submandibular gland (Supplementary Fig. S2B).

## Discussion

Cannabis users frequently report both dry mouth and dry eye as a side-effect. Several studies have examined aspects of how the cannabinoid signaling system might regulate salivation but no study to date has investigated this systematically. This is partly because traditional methods to study salivation in mouse models—and thereby take advantage of cannabinoid-related transgenic models—have, as a rule, involved invasive methods such as cannulation. To facilitate studies of cannabinoid regulation of salivation in mice, we developed a simple non-invasive method to measure basal salivation. Our chief findings are that THC reduces salivation in both males and females in a CB1-dependent manner and that CB1 receptors reside chiefly in cholinergic axons innervating the submandibular gland. We also found evidence that lipid signaling molecules mediate the reduction in salivation since deletion or inhibition of the NAE-metabolizing enzyme FAAH reduces salivation, while FAAH inhibitors had no effect in CB1 knockouts. FAAH protein is expressed within acinar but not ductal epithelial cells in the SMG. In lipidomic findings, FAAH knockout mice have increased levels of NAEs, including anandamide, and decreases in *N*-acyl glycines, 2-LG and *N*-arachidonoyl taurine among other changes.

**Figure 8:**
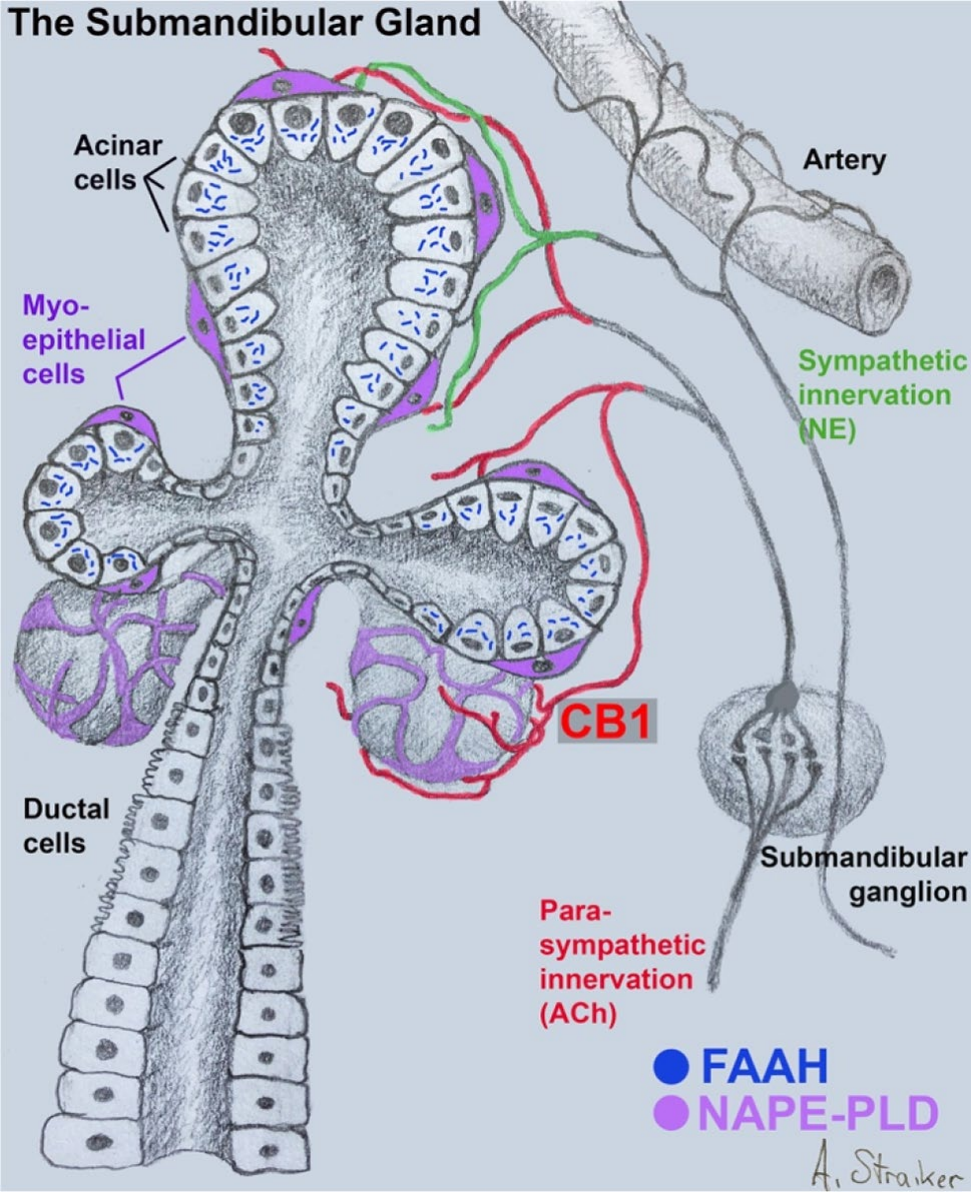
CB1 signaling system in submandibular gland. Schematic of submandibular gland showing acinar and ductal epithelial cells as well as enveloping myoepithelial cells (violet). CB1 receptors (red) are seen on the axons of parasympathetic neurons originating in the submandibular ganglion. FAAH, restricted to acinar cells, metabolizes acylethanolamines, including anandamide. NAPE-PLD likely resides in myoepithelial cells.

A reduction in acetylcholine release by cannabinoids was demonstrated in an elegant study by McConnell et al.^23^ showing that THC reduced basal salivary flow and the production of acetylcholine, but did not affect pilocarpine- or acetylcholine-stimulated salivary flow. Our findings indicate that key components of an intact cannabinoid signaling system are present in the submandibular gland. Our results are consistent with the following model of cannabinoid regulation of salivation: CB1 receptors are expressed on the axons of cholinergic neurons that innervate the submandibular gland; these CB1 receptors are activated by endogenous lipids, reducing acetylcholine release and, as a result, basal salivation. Endogenous lipids are then metabolized by FAAH that is expressed in acinar cells (schematized in Fig. 8). In this model, the effects of cannabis on salivation occur via THC activation of the CB1 signaling system in the submandibular gland. We find that CBD alone is without effect on salivation, but that CBD interferes with THC’s effects on salivation in a concentration-dependent manner. In the context of salivation, CBD reverses the effects of 4 mg/kg of THC with an IC50 of 4.0 mg/kg. This is consistent with reports that CBD antagonizes CB1 receptor activation, likely as a negative allosteric modulator^32,41^. We have found that this has functional physiological consequences in vivo both here and in countering the effects of THC on ocular pressure^9^, but by testing a range of concentrations we have learned that in vivo CBD is roughly equipotent as an inhibitor of THC.

In contrast to several published studies, we do not find evidence that CB2 receptor activation alters salivation acutely, i.e. 1 h after treatment. Kopach et al.^24^, Prestifillipo et al.^17^, and Rettori et al.^42^ each offered pharmacological evidence for CB2 receptor regulation of salivation. We have previously shown that several nominally selective CB2 agonists/antagonists (JWH15/AM630) also work well at CB1 receptors^43^. Each study also buttressed their findings with immunohistochemical analysis, but none were validated with the use of knockout mice.

Our finding that CB1 protein expression is chiefly neuronal/ axonal in nature contrasts with previous studies that reported CB1 protein in multiple cell types including acini and ductal epithelial cells^17,21,24^. Because those studies made use of rat^16,24^, or piglet^21^, neither was in a position to validate expression patterns in knockout animals, though it is possible that CB1 expression varies by species. Our immunohistochemical findings of CB1 and FAAH expression within the submandibular gland, and NAPE-PLD in myoepithelial cells, suggest the following circuit: NAEs are synthesized locally in myoepithelial cells, then act on CB1 receptors expressed in parasympathetic axons, after which they are broken down locally in non-ductal acinar cells (Fig. 8). The acinar cells would effectively act as a sump for stray/excess NAEs. The absence of an effect of a MAGL blocker on salivation as well as MAGL protein expression using immunohistochemistry is consistent with a primary role for NAEs over 2-AG in this system.

Our study was restricted to basal salivation and submandibular gland expression. Salivation is under complex control, regulated not only by parasympathetic and sympathetic inputs but also by inputs deriving from taste, smell and other sensory modalities. Some of this reflexive salivation occurs through the parotid gland, which adjoins the submandibular gland and has been reported to express functional cannabinoid receptors^20^, but was not the subject of the present study.

In our lipidomic analysis of FAAH KO mice vs. wild type controls, we saw expected increases in *N*-acyl ethanolamines (NAEs) and declines in *N*-acyl glycines (NAGlys) but some notable differences relative to findings in other tissues. Interestingly, we found that levels of three NAEs, anandamide, LEA and DHEA, were increased, while the levels of SEA were decreased and only OEA remained unaltered. This suggests that the longer, more unsaturated fatty acid ethanolamines, alone, in combination, or their metabolites, may be more likely to serve as the endogenous messengers mediating the effects of CB1 in the salivary gland. While in the CNS FAAH inhibitors generally raise NAEs as a group^39^, we have shown that this is not always the case in peripheral tissues, such as ileum and colon, where some NAEs either see no changes or even declines with FAAH inhibition^44^. This may be due to lower levels of FAAH expression in some peripheral structures compared to the CNS^45^ or may be an indication of more complex NAE metabolism in peripheral tissues. As noted above, we also saw substantial reductions in *N*-acyl glycines in the FAAH KO SMG. This family of lipids appears to require FAAH for synthesis^46^. Broadly speaking, there were fewer overall differences in the SMG lipidome relative to other tissues. For instance we have previously reported changes in *N*-acyl serines and 2-acyl glyerols in eye of FAAH KO^7^. With a few exceptions such as alterations in *N*-acyl taurines, the chief consequences of FAAH deletion were changes in NAEs and NAGlys. The role, if any, for these lipids is not always known, but some of these acyl species may be endogenous ligands at other targets. *N*-arachidonoyl glycine (NAGly), for example, is implicated as an agonist for the cannabinoid-related receptor GPR18^7,47^.

One attraction of examining CB1 regulation of both salivation and lacrimation is to allow a comparison across two related exocrine glands. Both tears and saliva are produced by exocrine glands under neuronal control—largely by autonomic inputs—and are partially innervated by the same brain stem nucleus. Given the reports of dry mouth and dry eye by cannabis users, we had predicted that both tearing and salivation would be reduced by CB1 receptor activation independent of sex. We have recently shown in mice that THC regulation of tearing is sex-dependent, with an inhibition in males but an increase in tearing in females^14^. Both effects are dependent on CB1 receptor activation. In contrast, CB1 receptor activation reduces salivation in mice in both sexes, a major difference between these two exocrine glands. The only sex-dependent difference we noted was in the time-course of URB597 effects, with females only reaching a significantly lower salivation at 3 h instead of the 1 h needed with males and a trending elevation in the level of FAAH protein in females. Therefore, while the two exocrine glands see some commonalities, particularly in that CB1 receptors on cholinergic inputs appear to reduce parasympathetic inputs, there are substantial qualitative differences between the two.

In summary, we have examined cannabinoid CB1 receptor regulation of salivation in mice. Though this is not the first study of this topic, this study benefits from the use of transgenic mice, either as gold-standard controls for immunohistochemistry or as controls for functional studies. Therefore, we can propose, with some confidence, a model for CB1 regulation of basal salivation via inhibition of parasympathetic neuronal inputs to the submandibular glands and a role for *N*-acyl ethanolamines and the metabolic enzyme FAAH. In contrast to our findings for tearing, this regulation of salivation is similar in males and females. Our results also suggest that the ‘cottonmouth’ frequently reported by cannabis users is due to THC activation of the CB1 receptors and that this is opposed, with similar potency, by CBD.

## Supplementary Information

Supplementary Figure S1.

Supplementary Figure S2.

Supplementary Figure S3.

## Data Availability

The datasets generated during and/or analysed during the current study are available from the corresponding author on reasonable request.

## References

[1] Proctor GB, Carpenter GH (2014). Salivary secretion: Mechanism and neural regulation. Monogr. Oral. Sci..

[2] Agostini BA (2018). How common is dry mouth? Systematic review and meta-regression analysis of prevalence estimates. Braz. Dent. J..

[3] Donaldson M, Goodchild JH (2018). A systematic approach to xerostomia diagnosis and management. Compend. Contin. Educ. Dent..

[4] Khanagar SB (2020). Age-related oral changes and their impact on oral health-related quality of life among frail elderly population: A review. J. Contemp. Dent. Pract..

[5] Patel R, Shahane A (2014). The epidemiology of Sjogren's syndrome. Clin. Epidemiol..

[6] Miller S (2016). Harnessing the endocannabinoid 2-arachidonoylglycerol to lower intraocular pressure in a murine model. Investig. Ophthalmol. Vis. Sci..

[7] Miller S (2016). Evidence for a GPR18 role in diurnal regulation of intraocular pressure. Investig. Ophthalmol. Vis. Sci..

[8] Miller S (2017). A GPR119 signaling system in the murine eye regulates intraocular pressure in a sex-dependent manner. Investig. Ophthalmol. Vis. Sci..

[9] Miller S, Daily L, Leishman E, Bradshaw H, Straiker A (2018). Delta9-tetrahydrocannabinol and cannabidiol differentially regulate intraocular pressure. Investig. Ophthalmol. Vis. Sci..

[10] Murataeva N (2015). Cannabinoid-induced chemotaxis in bovine corneal epithelial cells. Investig. Ophthalmol. Vis. Sci..

[11] Murataeva N (2019). Evidence for a GPR18 role in chemotaxis, proliferation, and the course of wound closure in the cornea. Cornea.

[12] Murataeva N (2019). Cannabinoid CB2R receptors are upregulated with corneal injury and regulate the course of corneal wound healing. Exp. Eye Res..

[13] Russo EB (2021). Survey of patients employing cannabigerol-predominant cannabis preparations: Perceived medical effects, adverse events, and withdrawal symptoms. Cannabis Cannabinoid Res..

[14] Thayer A (2020). THC regulates tearing via cannabinoid CB1 receptors. Investig. Ophthalmol. Vis. Sci..

[15] Dartt DA (2009). Neural regulation of lacrimal gland secretory processes: Relevance in dry eye diseases. Prog. Retin. Eye Res..

[16] Korchynska S (2019). GPR55 controls functional differentiation of self-renewing epithelial progenitors for salivation. JCI Insight..

[17] Prestifilippo JP (2006). Inhibition of salivary secretion by activation of cannabinoid receptors. Exp. Biol. Med. (Maywood).

[18] Prestifilippo JP, Fernandez-Solari J, Medina V, Rettori V, Elverdin JC (2009). Role of the endocannabinoid system in ethanol-induced inhibition of salivary secretion. Alcohol Alcohol..

[19] Prestifilippo JP (2013). Endocannabinoids mediate hyposalivation induced by inflammogens in the submandibular glands and hypothalamus. Arch. Oral. Biol..

[20] Busch L, Sterin-Borda L, Borda E (2004). Expression and biological effects of CB1 cannabinoid receptor in rat parotid gland. Biochem. Pharmacol..

[21] Pirino C (2018). The presence and distribution of cannabinoid type 1 and 2 receptors in the mandibular gland: The influence of different physical forms of diets on their expression in piglets. J. Anim. Physiol. Anim. Nutr. (Berl.).

[22] Fernandez-Solari J, Prestifilippo JP, Ossola CA, Rettori V, Elverdin JC (2010). Participation of the endocannabinoid system in lipopolysaccharide-induced inhibition of salivary secretion. Arch. Oral. Biol..

[23] McConnell WR, Dewey WL, Harris LS, Borzelleca JF (1978). A study of the effect of delta 9-tetrahydrocannabinol (delta 9-THC) on mammalian salivary flow. J. Pharmacol. Exp. Ther..

[24] Kopach O (2012). Cannabinoid receptors in submandibular acinar cells: Functional coupling between saliva fluid and electrolytes secretion and Ca2+ signalling. J. Cell Sci..

[25] Ledent C (1999). Unresponsiveness to cannabinoids and reduced addictive effects of opiates in CB1 receptor knockout mice. Science.

[26] Cravatt BF (2001). Supersensitivity to anandamide and enhanced endogenous cannabinoid signaling in mice lacking fatty acid amide hydrolase. Proc. Natl. Acad. Sci. U. S. A..

[27] Grim TW (2017). Apparent CB1 receptor rimonabant affinity estimates: Combination with THC and synthetic cannabinoids in the mouse in vivo triad model. J. Pharmacol. Exp. Ther..

[28] Hu SS (2010). Architecture of cannabinoid signaling in mouse retina. J. Comp. Neurol..

[29] Moussa-Tooks AB (2020). Long-term aberrations to cerebellar endocannabinoids induced by early-life stress. Sci. Rep..

[30] Leishman E, Kunkler PE, Hurley JH, Miller S, Bradshaw HB (2019). Bioactive lipids in cancer, inflammation and related diseases: Acute and chronic mild traumatic brain injury differentially changes levels of bioactive lipids in the CNS associated with headache. Adv. Exp. Med. Biol..

[31] Sain NM, Liang A, Kane SA, Urban MO (2009). Antinociceptive effects of the non-selective cannabinoid receptor agonist CP 55,940 are absent in CB1(−/−) and not CB2(−/−) mice in models of acute and persistent pain. Neuropharmacology.

[32] Straiker A, Dvorakova M, Zimmowitch A, Mackie K (2018). Cannabidiol inhibits endocannabinoid signaling in autaptic hippocampal neurons. Mol. Pharmacol..

[33] Roy J, Watson JE, Hong IS, Fan TM, Das A (2018). Antitumorigenic properties of omega-3 endocannabinoid epoxides. J. Med. Chem..

[34] Ghanbari MM, Loron AG, Sayyah M (2021). The omega-3 endocannabinoid docosahexaenoyl ethanolamide reduces seizure susceptibility in mice by activating cannabinoid type 1 receptors. Brain Res. Bull..

[35] Wu CS (2014). Long-term consequences of perinatal fatty acid amino hydrolase inhibition. Br. J. Pharmacol..

[36] Gulyas AI (2004). Segregation of two endocannabinoid-hydrolyzing enzymes into pre- and postsynaptic compartments in the rat hippocampus, cerebellum and amygdala. Eur. J. Neurosci..

[37] Nyilas R (2008). Enzymatic machinery for endocannabinoid biosynthesis associated with calcium stores in glutamatergic axon terminals. J. Neurosci..

[38] Carey LM (2016). A pro-nociceptive phenotype unmasked in mice lacking fatty-acid amide hydrolase. Mol. Pain..

[39] Leishman E (2016). Broad impact of deleting endogenous cannabinoid hydrolyzing enzymes and the CB1 cannabinoid receptor on the endogenous cannabinoid-related lipidome in eight regions of the mouse brain. Pharmacol. Res..

[40] Straiker A (2009). Monoacylglycerol lipase limits the duration of endocannabinoid-mediated depolarization-induced suppression of excitation in autaptic hippocampal neurons. Mol. Pharmacol..

[41] Laprairie RB, Bagher AM, Kelly ME, Denovan-Wright EM (2015). Cannabidiol is a negative allosteric modulator of the cannabinoid CB1 receptor. Br. J. Pharmacol..

[42] Rettori V (2007). Endocannabinoids in TNF-alpha and ethanol actions. NeuroImmunoModulation.

[43] Murataeva N, Mackie K, Straiker A (2012). The CB2-preferring agonist JWH015 also potently and efficaciously activates CB1 in autaptic hippocampal neurons. Pharmacol. Res..

[44] Fichna J (2014). Selective inhibition of FAAH produces antidiarrheal and antinociceptive effect mediated by endocannabinoids and cannabinoid-like fatty acid amides. Neurogastroenterol. Motil..

[45] Cravatt BF (2004). Functional disassociation of the central and peripheral fatty acid amide signaling systems. Proc. Natl. Acad. Sci. U. S. A..

[46] Bradshaw HB (2009). The endocannabinoid anandamide is a precursor for the signaling lipid *N*-arachidonoyl glycine by two distinct pathways. BMC Biochem..

[47] McHugh D (2010). *N*-arachidonoyl glycine, an abundant endogenous lipid, potently drives directed cellular migration through GPR18, the putative abnormal cannabidiol receptor. BMC Neurosci..

