# Supplementary material for: Cannabinoid CB1 receptors regulate salivation
Source: Sci Rep. 2022 Aug 19;12:14182. doi: 10.1038/s41598-022-17987-2 (PMC9391487; doi:10.1038/s41598-022-17987-2)

**Supplementary Figure 1**

**Supplementary Figure 1. FAAH protein in male vs. female submandibular gland.** A protein band is detected at the expected molecular weight (~63kda). Bottom panel shows total protein.


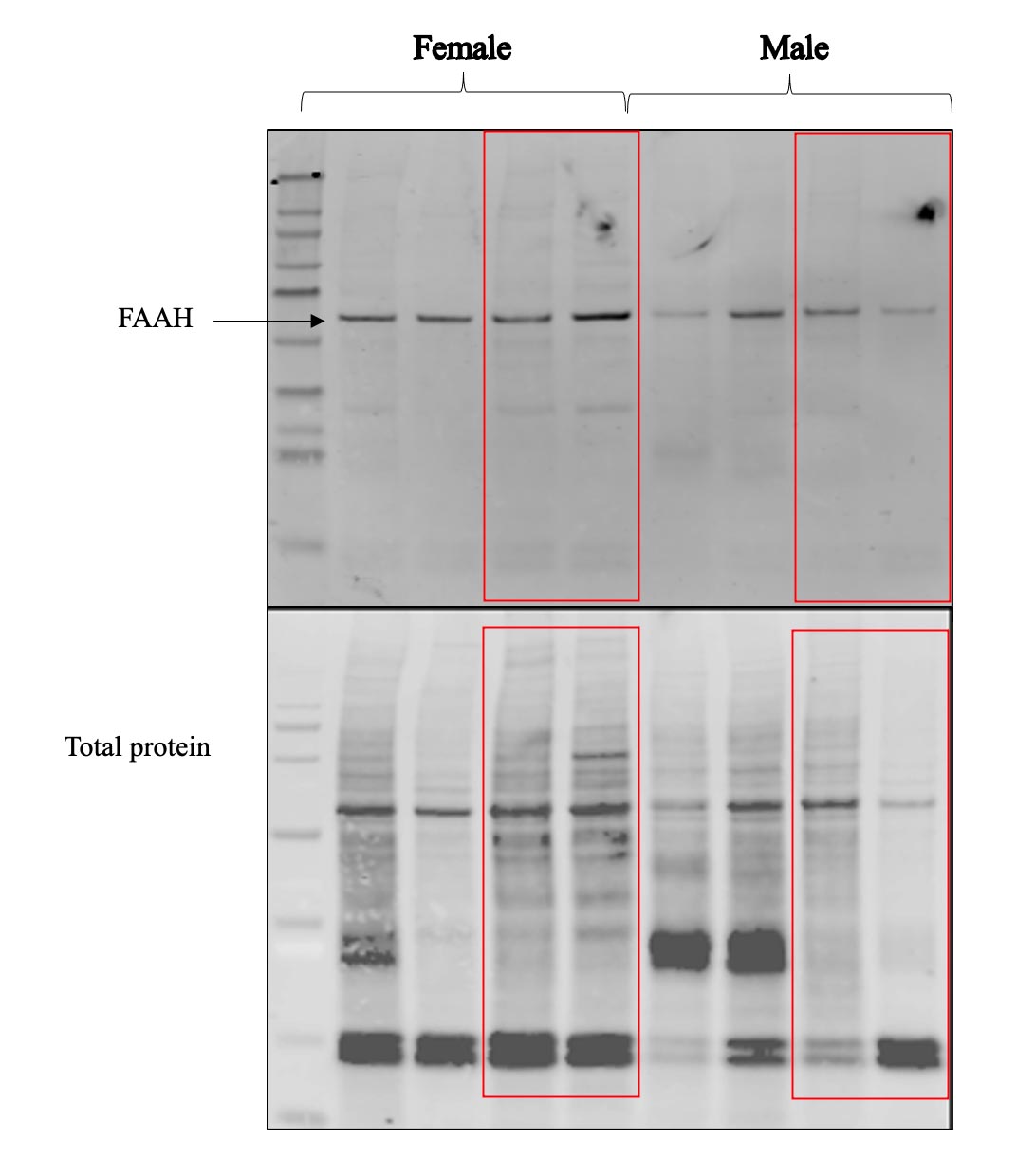

Supplement: Supplementary file 1 — Supplementary Figure S1. [file 41598_2022_17987_MOESM1_ESM.docx]
