# Supplementary material for: Cannabinoid CB1 receptors regulate salivation
Source: Sci Rep. 2022 Aug 19;12:14182. doi: 10.1038/s41598-022-17987-2 (PMC9391487; doi:10.1038/s41598-022-17987-2)

**Supplementary Figure 2**

**Figure S2. MAGL blocker JZL184 does not alter salivation in male or female mice and MAGL protein expression is not seen in submandibular gland.** A) Mice were treated with JZL184 (8mg/kg). Salivation was measured before treatment and again one hour after treatment. NS by paired t-test. B) Using immunohistochemistry, we did not detect MAGL (green) expression in SMG. Phalloidin (red) and DAPI (blue) were used to identify gross SMG structure. Images processed using Adobe Photoshop vsn. 21.2 and FIJI (vsn 2.3.0/1.53q, available at https://imagej.net/Fiji/downloads).


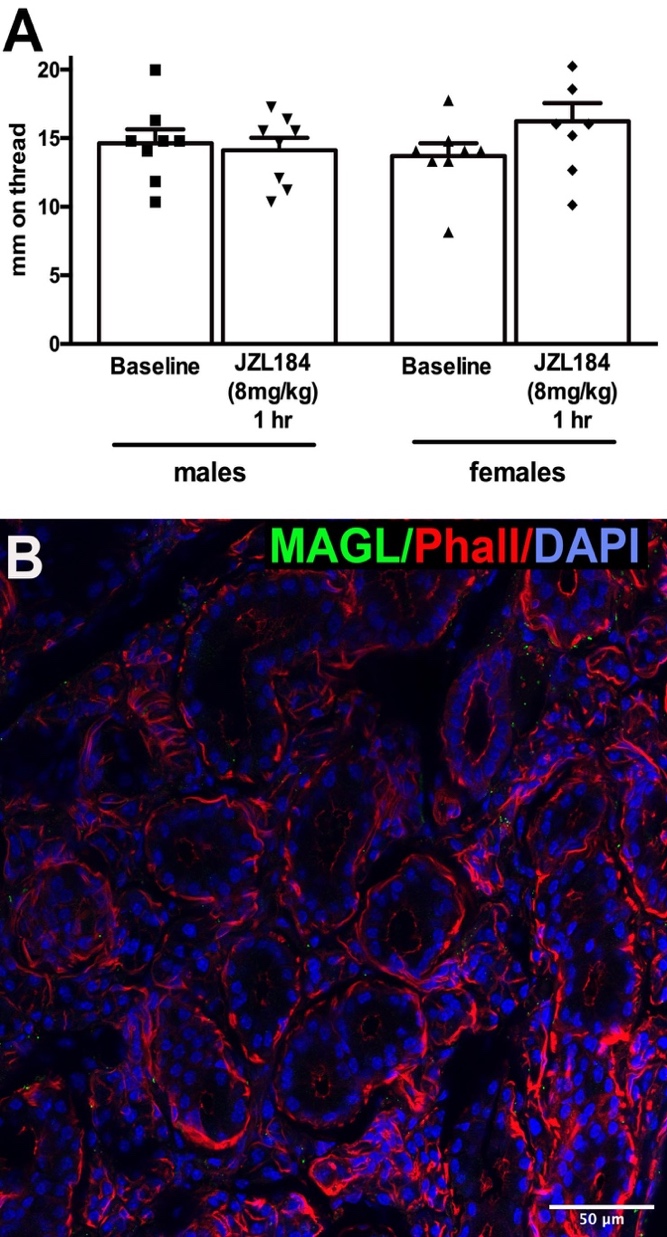

Supplement: Supplementary file 2 — Supplementary Figure S2. [file 41598_2022_17987_MOESM2_ESM.docx]
