# Supplementary material for: Cannabinoid CB1 receptors regulate salivation
Source: Sci Rep. 2022 Aug 19;12:14182. doi: 10.1038/s41598-022-17987-2 (PMC9391487; doi:10.1038/s41598-022-17987-2)

**Figure S3. NAPE-PLD protein is expressed in myoepithelial cells of submandibular gland.** A-B) Using immunohistochemistry in mouse submandibular gland we observed myoepithelial cell-like (MEC) staining for NAPE-PLD (green). Phalloidin in red. C) In rat SMG we observed substantial NAPE-PLD (green) overlap with smooth muscle actin (red), a marker for MECs. Phalloidin in blue. Images processed using Adobe Photoshop vsn. 21.2 and FIJI (vsn 2.3.0/1.53q, available at https://imagej.net/Fiji/downloads). Scale bar: 50um


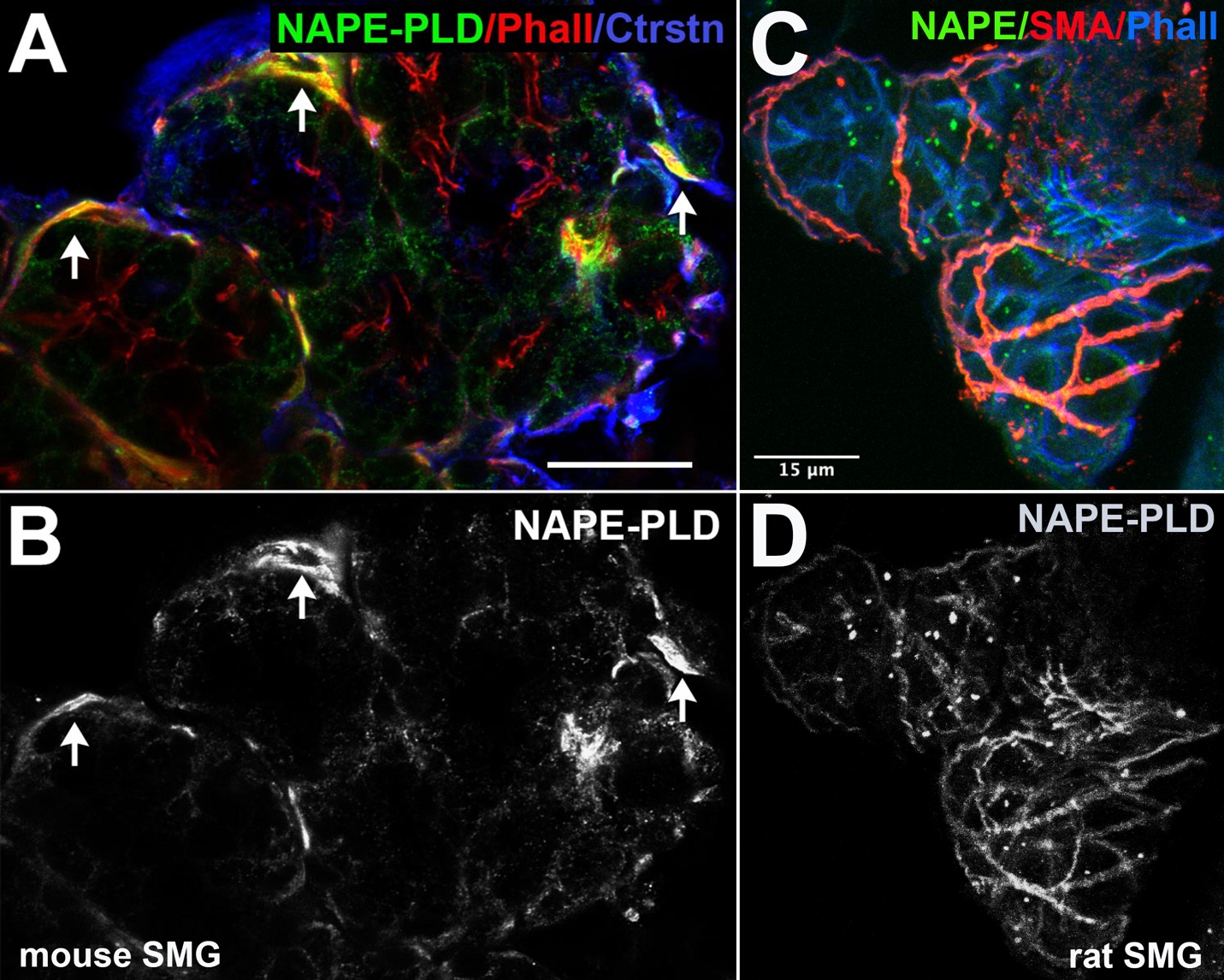

Supplement: Supplementary file 3 — Supplementary Figure S3. [file 41598_2022_17987_MOESM3_ESM.docx]
